# Ceftazidime-avibactam for multidrug-resistant gram-negative infections: outcomes and timing of initiation across 22 U.S. medical centers

**DOI:** 10.1128/aac.00268-26

**Published:** 2026-05-15

**Authors:** Ashlan J. Kunz Coyne, Chloe Judd, Kristen Lucas, Mohammed Al Musawa, Sean Van Helden, Dana Holger, Ali Althubyani, Christine Yost, Sydney VanDorf, Ryan Mynatt, Wesley D. Kufel, Jillian Hayes, Connor R. Deri, Rebekah H. Wrenn, Cara N. Slaton, Taylor Morrisette, Melisa Davis, M. Gabriela Cabanilla, Elizabeth Shald, Bryan P. White, Joseph Sassine, James Truong, Joshua Rosenberg, Justin Andrade, John Cerenzio, Natalia Medvedeva, David Ha, Taryn A. Eubank, Kevin W. Garey, Mark Biagi, Michaela Todd, S. Lena Kang-Birken, Caleb Rux, Kyle C. Molina, Christopher A. Jankowski, Kimberly C. Claeys, Veena Venugopalan, Michael P. Veve, Carolina Orzol, Mirna Eshaya, Kaylee E. Caniff, Callan Bleick, Michael J. Rybak

**Affiliations:** 1 Department of Pharmacy Practice, Anti-Infective Research Laboratory, Eugene Applebaum College of Pharmacy and Health Sciences, Wayne State University 2954, Detroit, Michigan, USA; 2 University of Kentucky College of Pharmacy 15511, Lexington, Kentucky, USA; 3 Barry and Judy Silverman College of Pharmacy, Nova Southeastern University 15478, Fort Lauderdale, Florida, USA; 4 Memorial Hospital West 23457, Pembroke Pines, Florida, USA; 5 Corewell Health William Beaumont University Hospital 21818, Royal Oak, Michigan, USA; 6 University of Kentucky HealthCare 177468, Lexington, Kentucky, USA; 7 State University of New York at Binghamton School of Pharmacy and Pharmaceutical Sciences 14787, Binghamton, New York, USA; 8 State University of New York Upstate Medical University 836016 , Syracuse, New York, USA; 9 Duke University Hospital 22957, Durham, North Carolina, USA; 10 Orlando Health 6246, Orlando, Florida, USA; 11 Department of Clinical Pharmacy and Outcomes Sciences, Medical University of South Carolina College of Pharmacy 2345, Charleston, South Carolina, USA; 12 Department of Pharmacy, University of New Mexico Hospital 21764, Albuquerque, New Mexico, USA; 13 OU Health University of Oklahoma Medical Center 6186, Oklahoma City, Oklahoma, USA; 14 NewYork-Presbyterian Queens Hospital, Queens, New York, USA; 15 Touro College of Pharmacy, The Brooklyn Hospital Center 12298, New York, New York, USA; 16 Stanford University School of Medicine 10624 , Stanford, California, USA; 17 University of Houston College of Pharmacy, Houston, Texas, USA; 18 UW Health SwedishAmerican Hospital, Rockford, Illinois, USA; 19 University of Illinois at Chicago 21725 , Rockford, Illinois, USA; 20 Santa Barbara Cottage Hospital 22854, Santa Barbara, California, USA; 21 Yale New Haven Hospital 25047, New Haven, Connecticut, USA; 22 Scripps Health 2697, San Diego, California, USA; 23 UF Health Jacksonville 21370 , Jacksonville, Florida, USA; 24 Department of Pharmacy Science and Health Outcomes Research, University of Maryland School of Pharmacy, Baltimore, Maryland, USA; 25 College of Pharmacy, University of Florida 3463, Gainesville, Florida, USA; 26 Henry Ford Hospital, Detroit, Michigan, USA; 27 Detroit Medical Center 2956, Detroit, Michigan, USA; Columbia University Irving Medical Center, New York, New York, USA

**Keywords:** ceftazidime/avibactam, multidrug-resistant, gram-negative infections, early initiation, carbapenem-resistant *Enterobacterales*, *Pseudomonas aeruginosa*, antimicrobial resistance, real-world evidence, rapid diagnostics

## Abstract

Ceftazidime-avibactam (CAZ-AVI) is a crucial treatment for multidrug-resistant (MDR) gram-negative infections; however, the impact of treatment timing and outcomes in real-world practice remains unclear. This study evaluated CAZ-AVI use across diverse U.S. centers, with emphasis on early initiation. We conducted a retrospective cohort study at 22 U.S. medical centers (2019–2025), including adults with MDR gram-negative infections who received CAZ-AVI ≥ 72 h. The primary outcome was composite clinical success, defined as 30-day survival, absence of microbiological recurrence, and resolution of fever and/or leukocytosis within 72 h of initiation. Early vs late initiation was assessed at 48 h. Classification and regression tree (CART) analysis was used to identify optimal thresholds. Among 613 patients (median age 60 years, 62.5% male, 57.1% admitted to the ICU within 24 h of index culture), the most common infection source was pneumonia (55.5%), and the most frequent pathogens were *Pseudomonas aeruginosa* (36.1%) and carbapenem-resistant *Enterobacterales* (34.7%). Composite clinical success occurred in 64.8%. CAZ-AVI initiation within 48 h was not significantly associated with improved outcomes (*P* = 0.064). Among patients who did not receive prior active antimicrobial therapy (*n* = 429), CART analysis identified a 42 h threshold. Initiation within 42 h was associated with higher clinical success (73.3% vs 63.4%, *P* = 0.046) and lower 30-day all-cause mortality (10.7% vs 19.1%, *P* = 0.030). In patients with pneumonia, the same threshold remained discriminatory, with improved clinical success and lower mortality. CAZ-AVI was effective for MDR gram-negative infections in real-world practice. Among patients without prior active therapy, initiation within 42 h was associated with improved outcomes, including in pneumonia.

## INTRODUCTION

Multidrug-resistant (MDR) gram-negative bacterial infections remain a persistent and escalating threat to public health in the United States. The Centers for Disease Control and Prevention (CDC) *Antibiotic Resistance Threats* report identifies carbapenem-resistant *Enterobacterales* (CRE) as an urgent priority, with attributable mortality rates of 20%–50% in bacteremic cases, and classifies MDR *Pseudomonas aeruginosa* as a serious concern (1). Collectively, these infections account for more than 35,000 deaths annually in the U.S., with estimated healthcare costs ranging from $7,352 for community-onset CRE to $66,973 for hospital-acquired infections (2, 3). They are most commonly associated with severe healthcare-associated infections, including bloodstream infections (BSI), hospital- and ventilator-associated bacterial pneumonia (HABP/VABP), complicated intra-abdominal infections (cIAI), and complicated urinary tract infections (cUTI). Resistance mechanisms driven by carbapenemases and extended-spectrum β-lactamases (ESBLs) have eroded the effectiveness of conventional antibiotics (4), underscoring the urgent need for therapeutic alternatives across diverse clinical settings.

Ceftazidime-avibactam (CAZ-AVI), a combination of ceftazidime with the novel β-lactamase inhibitor avibactam, has emerged as a critical option against MDR gram-negative infections. Initially approved by the U.S. Food and Drug Administration for cIAI and cUTI in adults with limited treatment options, its indications were later expanded to HABP/VABP (5). CAZ-AVI restores activity against CRE, ESBL-producers, and ceftazidime-resistant *P. aeruginosa* by inhibiting β-lactamases from classes A, C, and some D, though it remains ineffective against metallo-β-lactamases (MBLs) (6). These properties position CAZ-AVI as a cornerstone therapy, particularly in settings where resistance to conventional agents is prevalent.

Timely initiation of appropriate antibiotic therapy is a decisive determinant of outcomes in severe infections. Landmark sepsis studies show that every hour of delay after shock onset or emergency-department presentation increases mortality, with risk rising linearly from the first hour onward (7–9). A systematic review of 70 prospective studies found that appropriate empiric therapy reduced mortality odds by ~40%, corresponding to a number-needed-to-treat of 10 (10). Within this context, real-world evidence suggests a similar benefit for CAZ-AVI. Initiation within 48 h has been associated with ~50% lower odds of composite clinical failure across MDR gram-negative infections (11). In bacteremia due to carbapenemase-producing *Klebsiella pneumoniae*, delays beyond 24 h significantly increased 30-day mortality, whereas initiation within 24 h conferred a survival advantage (12). Comparable benefit has been observed in high-risk groups such as kidney-transplant recipients, where CAZ-AVI within 48 h was the strongest predictor of clinical cure and was associated with markedly reduced 30-day mortality (13). Although optimal thresholds may vary by infection type and diagnostic turnaround, the evidence consistently indicates that CAZ-AVI should be initiated as early as possible when MDR gram-negative infection is suspected or confirmed.

Clinical use of CAZ-AVI has expanded rapidly, driven by shortages of alternative agents such as ceftolozane–tazobactam and improvements in susceptibility testing. A systematic review reported that CAZ-AVI was used in 23% of 1,926 patients with carbapenem-resistant infections across global surveillance programs from 2015 to 2020 (14). More recently, a multicenter U.S. study reported a 52% clinical success rate with CAZ-AVI in patients with MDR *P. aeruginosa* pneumonia or BSI, with treatment-emergent resistance occurring in 23% of cases, highlighting the need for susceptibility testing and vigilance for resistance development (15). A 2025 meta-analysis further documented rising CAZ-AVI resistance globally, particularly among *K. pneumoniae* via *K. pneumoniae* carbapenemase (KPC) production and *P. aeruginosa* through efflux mechanisms, with higher prevalence outside the U.S. (16, 17). These trends, coupled with limited large-scale data on optimal timing and real-world outcomes, highlight the need for comprehensive studies to guide CAZ-AVI use in diverse clinical settings.

This study addresses these gaps by evaluating real-world outcomes of CAZ-AVI in 613 patients across 22 U.S. medical centers, with a focus on the impact of time to therapy initiation. The large, geographically diverse cohort provides a robust platform to inform optimal management of MDR gram-negative infections in the current therapeutic landscape.

## MATERIALS AND METHODS

### Study design

We conducted a retrospective, multicenter cohort study across 22 U.S. medical centers, representing all four geographic regions, to evaluate outcomes with CAZ-AVI in suspected or confirmed gram-negative infections. The primary comparison was between patients achieving composite clinical success and those experiencing failure, with timing of CAZ-AVI initiation (early vs late) assessed as a potentially modifiable risk factor. Data were collected between 1 June 2019 and 10 February 2025. Institutional Review Board approval was obtained at each site. A small subset of patients included in this cohort (up to 30 individuals) was also included in a previously published study (15); however, that study addressed a distinct research question and involved different analyses and outcomes.

The primary focus was the effect of CAZ-AVI initiation within vs beyond a prespecified 48 h threshold from the index culture. An exploratory classification and regression tree (CART) analysis was planned to identify an alternative time threshold if the 48 h cutoff was not significant (see Statistical Analysis). Data were managed in REDCap (Wayne State University) (18), with site collaborators entering data and investigators reviewing submissions for completeness. To promote consistency across sites, all participating investigators were provided with a standardized study protocol containing prespecified definitions for study variables and outcomes. In addition, the REDCap data collection instrument incorporated variable-level definitions that were available to site investigators in real time during data entry.

### Patients

Eligible patients were adults (≥18 years) hospitalized with suspected or confirmed gram-negative infections who received ≥72 continuous hours of CAZ-AVI. For infections caused by New Delhi metallo-β-lactamase (NDM)-producing organisms, patients were included if CAZ-AVI was administered in combination with aztreonam or with another agent demonstrating *in vitro* activity against the isolate. Exclusion criteria included transfer from an outside facility with positive gram-negative cultures, pregnancy, incarceration, or repeat CAZ-AVI courses within 60 days of the index culture.

### Variables and data collection

Data were extracted retrospectively from the electronic medical record after patient discharge. Collected variables included demographics, medical history, comorbidity burden (Charlson Comorbidity Index [19]), and severity of illness (Acute Physiology and Chronic Health Evaluation II [APACHE II] [20] and Sequential Organ Failure Assessment [SOFA] [21]), using the most severe values within 24 h of index culture.

Body mass index (BMI) was calculated as weight in kilograms divided by height in meters squared and categorized per CDC guidelines (22): underweight (<18.5 kg/m²), normal weight (18.5–24.9 kg/m²), overweight (25–29.9 kg/m²), and obese (≥30 kg/m²). Obesity was further subclassified into class 1 (30–34.9 kg/m²), class 2 (35–39.9 kg/m²), and class 3 (≥40 kg/m²).

Patients were classified as immunosuppressed if they met any of the following criteria: neutropenia (absolute neutrophil count [ANC] or white blood cell count <500 cells/µL), diagnosis of leukemia or lymphoma, AIDS (CD4 <200 cells/µL or AIDS-defining illness), prior splenectomy (functional or surgical), receipt of a solid organ or bone marrow transplant within 90 days, cytotoxic chemotherapy within 90 days, or treatment with high-dose corticosteroids (>40 mg/day prednisone equivalent for ≥14 days).

Additional variables included MDR risk factors, laboratory data, microbiological findings (e.g., organism identification and minimum inhibitory concentration [MIC]), infection source, and treatment-related details such as CAZ-AVI dose, duration, use of combination therapy, and prior active therapies. Combination therapy was defined as ≥24 h of overlap between CAZ-AVI and another active antibiotic. Consolidation of therapy was defined as discontinuation of one or more concurrent antimicrobial agents, with continuation of CAZ-AVI as definitive treatment for a confirmed mono- or polymicrobial infection. Timing metrics, including hours from index culture to Gram stain, organism identification, and MIC reporting, were also recorded.

Microbiological testing followed Clinical and Laboratory Standards Institute (CLSI) guidelines (23), using broth microdilution or automated systems per site protocol. Carbapenemase production was identified by phenotypic or molecular assays, as available. Multidrug-resistant *P. aeruginosa* was defined as non-susceptibility (intermediate or resistant) to at least one agent in three or more antibiotic categories, per CLSI breakpoints (23). Difficult-to-treat resistant (DTR) *P. aeruginosa* was defined as intermediate or resistant to all of the following antibiotics, based on MIC or other susceptibility methods exceeding current CLSI breakpoints: cefepime, ceftazidime, piperacillin/tazobactam, aztreonam, meropenem, imipenem/cilastatin, levofloxacin, and ciprofloxacin.

In polymicrobial infections, outcomes were attributed to the primary gram-negative pathogen based on clinical relevance and culture predominance, as determined by site investigators. Repeat cultures and susceptibility testing for emergent CAZ-AVI resistance (MIC ≥16 µg/mL) were performed at the discretion of each site, with no standardized schedule for frequency or timing.

### Outcomes

The primary outcome was composite clinical success, defined as all the following: (i) the absence of all-cause mortality within 30 days of index culture, (ii) the absence of microbiological recurrence within 30 days of the first CAZ-AVI dose, and (iii) resolution of fever and/or leukocytosis within 72 h of initiation. Leukocytosis was defined as white blood cell (WBC) > 10.8 × 10³/µL, assessed at or after 72 h following CAZ-AVI initiation using the first available value within a defined post-72 h window, and categorized as: resolution to normal range (4.0–10.8 × 10³/µL), reduction from baseline but not normalized, elevation due to causes unrelated to the index infection (e.g., *Clostridioides difficile* infection [CDI], surgery), persistent infection-related elevation, or no leukocytosis at baseline. Fever was defined as temperature >38°C, with outcomes assessed using the same approach, categorized as: resolution (≤38°C), reduction but not normalized, persistence due to causes unrelated to the index infection (e.g., drug fever), persistent infection-related fever, or no fever at baseline. Secondary outcomes included individual components of the composite, infection-related mortality, emergent CAZ-AVI resistance (MIC ≥16 µg/mL) up to 30 days during or after treatment, hospital length of stay, 30- and 60-day infection-related readmissions, and antibiotic-related adverse events (e.g., CDI, nephrotoxicity per Kidney Disease: Improving Global Outcomes [KDIGO] criteria [24]). Infection-related mortality was defined as death within 30 days of index culture attributable to MDR gram-negative infection, based on clinical signs (e.g., persistent fever, leukocytosis, and positive cultures) or sepsis-related organ failure, excluding causes unrelated to the index infection.

### Subgroups and statistical analysis

Bivariate analyses were conducted to compare baseline characteristics, clinical course, and treatment parameters between early and late CAZ-AVI initiation groups, using Chi-squared or Fisher’s exact tests for categorical variables and Student’s *t*-test or Mann–Whitney *U* test for continuous variables, with significance defined as *P* < 0.05. Multivariable binary logistic regression with backward stepwise (Wald) elimination was applied to identify independent predictors of clinical success, incorporating variables with *P* ≤ 0.10 in bivariate analyses. Candidate covariates included colonization with resistant organisms, age, body mass index categorized per CDC guidelines, APACHE II and SOFA scores, comorbidities such as acute kidney injury, chronic obstructive pulmonary disease, heart failure, and myocardial infarction, race (African American or Caucasian), prior active therapy, presumptive infection source (pneumonia or urinary tract), and hours from index culture to CAZ-AVI initiation.

Inverse Probability of Treatment Weighting was used to adjust the early vs late comparison for potential confounders, including prior infection with resistant organisms, blood culture source, resistance phenotypes, prior active therapy, infectious disease consultation, and appropriate dosing as listed in the package insert. To evaluate the association between time from index culture to CAZ-AVI initiation and composite clinical success, a CART analysis was performed using recursive partitioning. The analysis was conducted among patients who did not receive active antimicrobial therapy prior to CAZ-AVI initiation. Patients were classified into early and late therapy groups based on the primary CART-derived time split, with additional candidate splits evaluated but not retained due to limited subgroup sizes. The CART-derived threshold of 42 h was used to define early vs late initiation for subsequent analyses. CART analyses were conducted using R version 4.4.3 (R Foundation for Statistical Computing, Vienna, Austria).

## RESULTS

### Patient demographics and baseline characteristics

A total of 613 patients were included, with a median age of 60 years (interquartile range [IQR] 47–69) and 62.5% male (Table 1). The cohort was racially diverse (45.7% Caucasian and 34.9% African American). Median BMI was 26.6 kg/m² (IQR 22.6–32.2), with 32.6% obese (BMI ≥30 kg/m²). Illness severity was high, with a median APACHE II score of 23 (IQR 16–28), SOFA score of 5 (IQR 2–8), and a Charlson Comorbidity Index of 2 (IQR 1–3). Immunosuppression was present in 17%, and MDR risk factors were common (89.7%), including prior antimicrobials (63.3%) and hospitalization (67.5%) within 90 days. Common comorbidities included diabetes (41.3%), heart failure (16.5%), and moderate-to-severe chronic kidney disease (16.2%). Over half (57.1%) required ICU admission within 24 h of index culture. The most common infection sources were pneumonia (55.5%), bloodstream (18.6%), and urinary tract (15%; Table 2).

**TABLE 1:** Baseline characteristics^*a*^^,^^*b*^^,^^*c*^

Parameter	Total	Success	Fail	*P*-value
	*n* = 613	*n* = 397	*n* = 216	
Age (years), median (IQR)	60 (47, 69)	60 (45.8, 69.3)	61 (51, 70)	**0.034**
Male	383 (62.5)	246 (62)	137 (63.4)	0.721
Race/ethnicity				
Caucasian	280 (45.7)	191 (48.1)	89 (41.2)	0.101
African American	214 (34.9)	128 (32.3)	86 (39.8)	0.06
Hispanic	59 (9.6)	42 (10.6)	17 (7.9)	0.277
Asian	9 (1.5)	6 (1.5)	3 (1.4)	0.904
Other	50 (8.2)	29 (7.3)	21 (9.7)	0.269
BMI (kg/m^2^), median (IQR)	26.6 (22.6, 32.2)	26 (22.1, 31.4)	28 (23.8, 32)	**0.008**
Obese (BMI ≥ 30 kg/m^2^)	200 (32.6)	119 (30)	81 (37.5)	0.058
Class 1	97 (15.8)	60 (15.1)	37 (17.1)	0.513
Class 2	55 (9)	24 (11.7)	31 (7.8)	**0.024**
Class 3	48 (7.8)	31 (7.8)	17 (7.9)	0.978
Admitted from				
Home	308 (50.2)	203 (51.1)	105 (48.6)	0.551
Nursing home/skilled nursing facility	160 (26.1)	103 (25.9)	57 (26.4)	0.905
Transfer from outside hospital	92 (15.1)	54 (13.6)	38 (17.6)	0.186
Referral from clinic	27 (4.4)	20 (5)	7 (3.2)	0.3
Long-term acute care	15 (2.4)	10 (2.5)	5 (2.3)	0.876
Inpatient rehab facility	10 (1.6)	6 (1.5)	4 (1.9)	0.751
Unhoused	1 (0.2)	1 (0.3)	0 (0)	0.46
Severity scores, median (IQR)				
APACHE II score	23 (16, 28)	22 (14.8, 26)	25 (19, 32)	**<0.001**
SOFA score	5 (2, 8)	5 (2, 7)	7 (5, 11)	**<0.001**
Charlson Comorbidity Index	2 (1, 3)	2 (1, 3)	1 (1, 4)	0.45
Immunosuppression	104 (17)	68 (17.1)	36 (16.7)	0.884
Neutropenia (ANC or WBC < 500)	34 (5.5)	23 (5.8)	11 (5.1)	0.717
Cytotoxic chemotherapy in preceding 90 days	34 (5.5)	23 (5.8)	11 (5.1)	0.717
Solid organ transplant in preceding 90 days	26 (4.2)	19 (4.8)	7 (3.2)	0.365
High-dose corticosteroids	23 (3.8)	12 (3)	11 (5.1)	0.198
Bone marrow transplant in preceding 90 days	4 (0.7)	2 (0.5)	2 (0.9)	0.535
Splenectomy (functional or surgical)	3 (0.5)	3 (0.8)	0 (0)	0.200
AIDS (CD4 count < 200 or AIDS-defining illness)	2 (0.3)	1 (0.3)	1 (0.5)	0.662
MDR risk factors	550 (89.7)	362 (91.2)	188 (87)	0.106
Hospitalization ≥ 48 h in 90 days before IC	414 (67.5)	272 (68.5)	142 (65.7)	0.484
Antimicrobials ≥ 24 h in 90 days before IC	388 (63.3)	258 (65)	130 (60.2)	0.239
Prior infection with resistant organisms	182 (29.7)	122 (30.7)	60 (27.8)	0.445
Admitted from NH or extended care facility	170 (27.7)	110 (27.7)	60 (27.8)	0.985
Colonization with resistant organisms	106 (17.3)	80 (20.2)	26 (12)	**0.011**
Surgery in 30 days before index culture	67 (10.9)	19 (9.2)	46 (11.6)	0.129
Chronic dialysis in 30 days before index culture	37 (6)	25 (6.3)	12 (5.6)	0.713
Home wound care	29 (4.7)	19 (4.8)	10 (4.6)	0.931
Home infusion (includes antibiotics)	18 (2.9)	12 (3)	6 (2.8)	0.864
Comorbid conditions				
Diabetes	253 (41.3)	168 (42.3)	85 (39.4)	0.476
Heart failure	101 (16.5)	56 (14.1)	45 (20.8)	**0.032**
Moderate to severe CKD	99 (16.2)	61 (15.4)	38 (17.6)	0.474
COPD	91 (14.8)	52 (13.1)	39 (18.1)	0.099
Cerebrovascular disease	86 (14)	53 (13.4)	33 (15.3)	0.512
Peripheral vascular disease	85 (13.9)	51 (12.8)	34 (15.7)	0.322
COVID-19	74 (12.1)	42 (10.6)	32 (14.8)	0.124
Acute kidney injury	71 (11.6)	39 (9.8)	32 (14.8)	0.065
Myocardial infarction	54 (8.8)	29 (7.3)	25 (11.6)	0.075
Asthma	44 (7.2)	27 (6.8)	17 (7.9)	0.624
Chronic dialysis	40 (6.5)	26 (6.5)	14 (6.5)	0.974
Dementia	32 (5.2)	20 (5)	12 (5.6)	0.783
Hemiplegia	29 (4.7)	20 (5)	9 (4.2)	0.627
Connective tissue disease	27 (4.4)	15 (3.8)	12 (5.6)	0.306
Tumor without metastasis	27 (4.4)	18 (4.5)	9 (4.2)	0.832
Moderate to severe liver disease	25 (4.1)	17 (4.3)	8 (3.7)	0.729
Tumor with metastasis	23 (3.8)	16 (4)	7 (3.2)	0.623
Cystic fibrosis	19 (3.1)	15 (3.8)	4 (1.9)	0.189
Leukemia	15 (2.4)	7 (1.8)	8 (3.7)	0.137
Peptic ulcer disease	12 (2)	9 (2.3)	3 (1.4)	0.453
Clostridium difficile-associated diarrhea	10 (1.6)	6 (1.5)	4 (1.9)	0.751
IV drug use	9 (1.5)	7 (1.8)	2 (0.9)	0.410
Lymphoma	8 (1.3)	6 (1.5)	2 (0.9)	0.542
Mild liver disease	7 (1.1)	7 (1.8)	0 (0)	**0.050**
HIV	6 (1)	4 (1)	2 (0.9)	0.364
AIDS (CD4 count < 200)	2 (0.3)	1 (0.3)	1 (0.5)	0.662

^*a*^ Data presented as *n *(%) unless otherwise specified.

^*b*^ IQR, interquartile range; BMI, body mass index; APACHE, Acute Physiology and Chronic Health Evaluation; SOFA, sequential organ failure assessment; ANC, absolute neutrophil count; WBC, white blood cell; AIDS, acquired immunodeficiency syndrome; IC, index culture collection; NH, nursing home; COPD, chronic obstructive pulmonary disease; CKD, chronic kidney disease; HIV, human immunodeficiency virus; IV, intravenous.

^*c*^ Boldface indicates that the *P*-value is significant.

**TABLE 2:** Clinical course and treatment characteristics^*d*^^,^^*e*^

Parameter	Total	Success	Fail	*P*-value
	*n* = 613	*n* = 397	*n* = 216	
Presumptive primary source of infection				
Pneumonia	340 (55.5)	202 (50.9)	138 (63.9)	**0.002**
UTI	92 (15)	68 (17.1)	24 (11.1)	**0.046**
Intraabdominal	51 (8.3)	35 (8.8)	16 (7.4)	0.54
Skin/skin structure	40 (6.5)	25 (6.3)	15 (6.9)	0.763
Abscess	18 (2.9)	15 (3.8)	3 (1.4)	0.093
Bone and joint	17 (2.8)	14 (3.5)	4 (1.9)	0.241
IV catheter	13 (2.1)	8 (2)	5 (2.3)	0.806
Endovascular	5 (0.8)	3 (0.8)	2 (0.9)	0.823
Invasive prosthetic device	5 (0.8)	5 (1.3)	0 (0)	0.098
CNS/vertebral abscess	2 (0.3)	2 (0.5)	0 (0)	0.295
Endocarditis	1 (0.2)	1 (0.3)	0 (0)	0.46
Other	13 (2.1)	10 (2.5)	3 (1.4)	0.354
Unknown	14 (2.3)	8 (2)	6 (2.8)	0.546
Culture source				
Sputum	235 (38.3)	144 (36.3)	91 (42.1)	0.154
Blood	114 (18.6)	70 (17.6)	44 (20.4)	0.405
Urine	82 (13.4)	61 (15.4)	21 (9.7)	**0.050**
Endotracheal aspirate	57 (9.3)	27 (6.8)	30 (13.9)	**0.004**
Bronchoalveolar lavage	48 (7.8)	28 (7.1)	20 (9.3)	0.331
Fluid	44 (7.2)	28 (7.1)	16 (7.4)	0.871
Wound	37 (6)	29 (7.3)	8 (3.7)	0.074
Tissue	28 (4.6)	24 (6)	4 (1.9)	**0.018**
Bone	6 (1)	6 (1.5)	0 (0)	0.069
Catheter tip	2 (0.3)	2 (0.5)	0 (0)	0.296
Other	12 (2)	9 (2.3)	3 (1.4)	0.453
Polymicrobial infection	308 (50.2)	197 (49.6)	106 (49.1)	0.897
Monomicrobial infection	305 (49.8)	196 (49.4)	109 (50.5)	0.796
Organisms targeted by CAZ/AVI^*a*^				
*Pseudomonas aeruginosa*	423 (36.1)	282 (37.3)	141 (33.3)	0.178
Mucoid *P. aeruginosa*	15 (3.5)	11 (6)	4 (4)	0.488
*Klebsiella pneumoniae*	188 (16)	114 (15.1)	74 (17.5)	0.273
*Escherichia coli*	67 (5.7)	47 (6.2)	20 (4.7)	0.283
*Proteus mirabilis*	54 (4.6)	37 (4.9)	17 (4)	0.493
*Enterobacter cloacae*	46 (3.9)	22 (5.5)	11 (5.1)	0.814
*Serratia marcescens*	31 (2.6)	17 (2.2)	14 (3.3)	0.273
*Providencia stuartii*	23 (2)	11 (1.5)	12 (2.8)	0.099
*Enterobacter aerogenes*	18 (1.5)	13 (1.7)	5 (1.2)	0.472
*Citrobacter freundii*	7 (0.6)	3 (0.4)	4 (0.9)	0.239
Other *Citrobacter* spp.	6 (0.5)	4 (0.5)	2 (0.5)	0.898
*Morganella morganii*	6 (0.5)	3 (0.4)	3 (0.7)	0.469
*Klebsiella oxytoca*	5 (0.4)	1 (0.1)	4 (0.9)	**0.039**
*Proteus vulgaris*	1 (0.1)	1 (0.1)	0 (0)	0.455
Other organisms isolated/treated during CAZ/AVI^*a*^				
*Enterococcus faecalis*	41 (3.5)	27 (3.6)	14 (3.3)	0.817
Yeast (*Candida*)	37 (3.2)	25 (3.3)	12 (2.8)	0.66
MRSA	31 (2.6)	20 (2.7)	11 (2.6)	0.951
*Enterococcus faecium*	30 (2.6)	22 (2.9)	8 (1.9)	0.288
CoNS	28 (2.4)	17 (2.2)	11 (2.6)	0.46
*Stenotrophomonas maltophilia*	24 (2)	11 (1.5)	13 (3.1)	0.059
*Achromobacter xylosoxidans*	9 (0.8)	6 (0.8)	3 (0.7)	0.875
*Streptococcus* spp.	8 (0.7)	4 (0.5)	4 (0.9)	0.402
MSSA	6 (0.5)	4 (0.5)	2 (0.5)	0.891
Other *Klebsiella*	6 (0.5)	4 (0.5)	2 (0.5)	0.891
Other *Pseudomonas*	4 (0.3)	2 (0.3)	2 (0.5)	0.554
Other *Stenotrophomonas*	3 (0.3)	1 (0.1)	2 (0.5)	0.268
*Anaerobe*	3 (0.3)	3 (0.4)	0 (0)	0.06
Other	36 (3.1)	26 (3.3)	9 (2.3)	0.362
Resistance phenotypes and carbapenemases^*b*^				
CRE	213 (34.7)	133 (33.5)	80 (37)	0.38
KPC	46 (7.5)	25 (6.3)	21 (9.7)	0.2
NDM	13 (2.1)	8 (2)	5 (2.3)	0.945
CTX-M	6 (1)	4 (1)	2 (0.9)	0.828
OXA-48-like	4 (0.7)	3 (0.8)	1 (0.5)	0.601
Unknown, but confirmed	62 (10.1)	43 (10.8)	19 (8.8)	0.182
DTR, *P. aeruginosa*	98 (16)	54 (13.6)	37 (17.1)	0.241
KPC	1 (0.2)	0 (0)	1 (0.5)	0.175
Unknown, but confirmed	4 (0.7)	2 (0.5)	2 (0.9)	0.535
MDR, *P. aeruginosa*	50 (8.2)	28 (7.1)	17 (7.9)	0.711
CTX-M	1 (0.2)	0 (0)	1 (0.5)	0.175
Unknown, but confirmed	3 (0.5)	2 (0.5)	1 (0.5)	0.945
CR-*P. aeruginosa*	314 (74.2)	205 (51.6)	109 (50.5)	0.781
None of the above	108 (17.6)	71 (17.9)	37 (17.1)	0.815
Combination therapies	104 (17)	62 (15.7)	42 (19.4)	0.239
Inhaled tobramycin	37 (6)	22 (5.5)	15 (6.9)	0.486
Inhaled colistin	8 (1.3)	6 (1.5)	2 (0.9)	0.542
Inhaled amikacin	2 (0.3)	2 (0.5)	0 (0)	0.269
Inhaled aztreonam	1 (0.2)	0 (0)	1 (0.5)	0.175
Aztreonam	23 (3.8)	14 (3.5)	9 (4.2)	0.69
Tobramycin	18 (2.9)	13 (3.3)	5 (2.3)	0.501
Amikacin	13 (2.1)	9 (2.3)	4 (1.9)	0.733
Gentamicin	5 (0.8)	2 (0.5)	3 (1.4)	0.244
Colistin	5 (0.8)	3 (0.8)	2 (0.9)	0.823
Ciprofloxacin, IV	4 (0.7)	1 (0.3)	3 (1.4)	0.095
Polymyxin	3 (0.5)	1 (0.3)	2 (0.9)	0.253
Tigecycline	3 (0.5)	2 (0.5)	1 (0.5)	0.945
Meropenem	3 (0.5)	1 (0.3)	2 (0.9)	0.253
Levofloxacin, IV	3 (0.5)	1 (0.3)	2 (0.9)	0.253
Cefepime	1 (0.2)	1 (0.3)	0 (0)	0.46
TMP-SMX, IV	1 (0.2)	1 (0.3)	0 (0)	0.46
Eravacycline	1 (0.2)	1 (0.3)	0 (0)	0.46
Other	53 (8.6)	36 (9.1)	17 (7.9)	0.614
ICU admissions				
One admission	328 (53.5)	193 (48.6)	135 (62.5)	**<0.001**
Two admissions	68 (11.1)	36 (9.1)	32 (14.8)	**0.030**
Three admissions	42 (6.9)	30 (7.6)	12 (5.6)	0.349
ICU within 24 h of index culture	350 (57.1)	205 (52.4)	145 (67.4)	**<0.001**
Active therapy before CAZ/AVI	175 (28.5)	104 (26.5)	71 (33)	0.091
Cefepime	54 (8.8)	30 (7.6)	24 (11.1)	0.138
Meropenem	43 (7)	23 (5.8)	20 (9.3)	0.108
Piperacillin-Tazobactam	26 (4.2)	20 (5)	6 (2.8)	0.185
Ceftolozane-Tazobactam	12 (2)	7 (1.8)	5 (2.3)	0.638
Tobramycin	12 (2)	9 (2.3)	3 (1.4)	0.453
Ertapenem	11 (1.8)	8 (2)	4 (1.9)	0.889
Amikacin	11 (1.8)	6 (1.5)	5 (2.3)	0.474
TMP-SMX	6 (1)	4 (1)	2 (0.9)	0.922
Ciprofloxacin	5 (0.8)	2 (0.5)	3 (1.4)	0.244
Levofloxacin	5 (0.8)	2 (0.5)	3 (1.4)	0.244
Gentamicin	5 (0.8)	4 (1)	1 (0.5)	0.474
Tigecycline	1 (0.2)	0 (0)	1 (0.5)	0.175
Ceftazidime	1 (0.2)	1 (0.3)	0 (0)	0.46
ID consult	578 (94.3)	376 (94.7)	202 (93.5)	0.544
Rationale for CAZ/AVI use				
No other active agent for infection	247 (40.3)	167 (42.1)	80 (37)	0.225
Double coverage for suspected CR	150 (24.5)	86 (21.7)	64 (29.6)	**0.028**
Consolidation of regimen	140 (22.8)	95 (23.9)	45 (20.8)	0.383
Antibiotic shortage	41 (6.7)	32 (8.1)	9 (4.2)	0.065
Lack of PO access	1 (0.2)	0 (0)	1 (0.5)	0.175
Other	99 (16.2)	61 (15.4)	38 (17.6)	0.474
CAZ/AVI treatment				
0.94 g every 12 h	23 (3.8)	11 (2.8)	12 (5.6)	0.083
0.94 g every 24 h	26 (4.2)	15 (3.8)	11 (5.1)	0.441
0.94 g every 48 h	14 (2.3)	10 (2.5)	4 (1.9)	0.597
1 g every 12 h	6 (1)	2 (0.5)	4 (1.9)	0.105
1 g every 48 h	4 (0.7)	1 (0.3)	3 (1.4)	0.095
1.25 g every 8 h	98 (16)	52 (13.1)	46 (21.3)	**0.008**
1.25 g every 12 h	7 (1.1)	3 (0.8)	4 (1.9)	0.222
1.25 g every 24 h	5 (0.8)	3 (0.8)	2 (0.9)	0.823
2.5 g every 6 h	2 (0.3)	2 (0.9)	0 (0)	0.055
2.5 g every 8 h	423 (69)	296 (74.6)	127 (58.8)	**<0.001**
2.5 g every 12 h	2 (0.3)	1 (0.3)	1 (0.5)	0.662
5 g every 8 h	3 (0.5)	3 (0.8)	0 (0)	0.200
Appropriate CAZ/AVI dose^*c*^	538 (87.8)	358 (90.2)	180 (83.3)	**0.014**
Hospital length of stay (days)	29.0 (13.5, 53.8)	27.7 (12.1, 55)	30.0 (15.8, 49.1)	0.333
Discharge disposition				
NH, skilled nursing facility, LTAC	208 (33.9)	160 (40.4)	48 (22.5)	**<0.001**
Home	191 (31.2)	168 (42.3)	23 (10.6)	**<0.001**
Morgue	117 (19.1)	19 (4.8)	98 (46.0)	**<0.001**
Hospice	57 (9.3)	22 (5.6)	35 (16.4)	**<0.001**
Rehabilitation center	36 (5.9)	27 (6.8)	9 (4.2)	0.196

^*a*^ The total number of identified organisms exceeds the total study population due to polymicrobial infections containing multiple organisms. Percentages were determined using the total number of isolates as the denominator (*n *= 1172).

^*b*^ Percentages are based on the number of patients.

^*c*^ Appropriateness determined by package insert directions for renal dose adjustments.

^*d*^ UTI, urinary tract infection; IV, intravenous; CNS, central nervous system; CAZ/AVI, ceftazidime/avibactam; MRSA, methicillin-susceptible *Staphylococcus aureus*; MSSA, methicillin-susceptible *Staphylococcus aureus*; CoNS, coagulase-negative *Staphylococcus aureus*; CRE, carbapenem-resistant *Enterobacterales*; DTR, difficult-to-treat; MDR, multidrug resistant; TMP-SMX, trimethoprim-sulfamethoxazole; ICU, intensive care unit; ID, infectious diseases; PO, oral; CR, carbapenem resistance; CrCl, creatinine clearance; NH, nursing home.

^*e*^ Boldface indicates that the *P*-value is significant.

A total of 1,172 isolates were identified, with *P. aeruginosa* predominant (36.1%, including 3.5% mucoid strains), followed by *K. pneumoniae* (16%; Table 2). Of the 613 patients, 31.7% had polymicrobial infections, and 34.7% had CRE isolated. Resistance phenotypes included CRE (72.9% among applicable isolates), DTR *P. aeruginosa* (16%), and MDR *P. aeruginosa* (8.2%). CAZ-AVI susceptibility was 88.4% overall (Table 3), with 96.8% for *P. aeruginosa* (97.1% for carbapenem-resistant *P. aeruginosa*), 78.5% for *Enterobacterales* (most isolates [82/153] were *K. pneumoniae* with 78.6% susceptibility), and 87.4% for CRE.

**TABLE 3:** Ceftazidime-avibactam susceptibility of index culture isolates^*a*^

	MIC (μg/mL)		Disk diffusion (mm)		Interpretation only		R total
Organism	S (≤8/4)	R (≥16/4)	S (≥21)	R (≤20)	S	R	*n* (%)
*Pseudomonas aeruginosa*	231	7	5	1	8	0	8 (31.5)
*Klebsiella pneumoniae*	82	24	2	0	8	1	25 (4.7)
*Citrobacter freundii*	5	0	0	0	0	0	0 (0)
Other *Citrobacter* spp.	3	0	0	0	0	0	0 (0)
*Enterobacter aerogenes*	6	0	0	0	1	0	0 (0)
*Enterobacter cloacae*	19	3	1	0	2	0	3 (8.3)
*Escherichia coli*	14	9	0	0	0	0	9 (39.1)
*Klebsiella oxytoca*	3	1	0	0	0	0	1 (25)
*Morganella morganii*	1	0	0	0	1	0	0 (0)
*Proteus mirabilis*	4	5	0	0	0	0	5 (55.6)
*Providencia stuartii*	2	1	0	0	0	0	1 (33.3)
*Serratia marcescens*	14	2	0	0	0	0	2 (12.5)
Total	384	52	8	1	20	1	54 (11.6)
Carbapenem-resistant *Pseudomonas aeruginosa*	224	7	5	0	4	0	7 (2.9)
Carbapenem-resistant Enterobacterales	127	19	3	0	9	1	20 (12.6)

^*a*^ MIC, minimum inhibitory concentrations; S, susceptible; R, resistant.

CAZ-AVI was initiated a median of 74 h (IQR 37–111) after index culture with a median duration of 8 days (IQR 6–14). Dosing was predominantly 2.5 g every 8 h (69%), with 87.8% appropriately adjusted for renal function. Combination therapy was used in 17%, most commonly with inhaled aminoglycosides (e.g., tobramycin, 6%). Prior active therapy was administered in 28.5%, primarily cefepime (8.8%) or meropenem (7%). Infectious disease consultation occurred in 94.3%.

Repeat susceptibility testing was performed in 199 patients (32.5%). Emergent CAZ-AVI resistance (MIC ≥16 µg/mL) was identified in 25 patients, corresponding to 12.6% of those tested and 4.1% of the overall cohort; 20% of these cases occurred in patients who received inappropriate dosing. Among patients with emergent resistance, 11 (44%) achieved composite clinical success. Resistance most frequently emerged in *P. aeruginosa* (20/25, 80%), with fewer cases observed in *K. pneumoniae* (2, 8%) and other Enterobacterales. Most of these infections were respiratory in origin (22, 88%), with fewer cases involving intra-abdominal (2, 8%) and skin and soft tissue sources (1, 4%).

Among patients with CRE, carbapenemase distribution included KPC (7.5%), OXA-48-like (0.7%), and NDM producers (2.1%), while 10.1% had confirmed carbapenem resistance without a defined mechanism (Table 2). Among the 13 patients with NDM-producing CRE, clinical success did not differ significantly between groups. Eight patients received combination therapy with aztreonam plus CAZ-AVI, of whom six achieved clinical success. Among the remaining patients, tigecycline or levofloxacin was used in combination with CAZ-AVI. NDM-producing isolates were recovered from a range of infection sources, most commonly respiratory (30.8%) and intra-abdominal (23.1%) infections.

Difficult-to-treat resistant *P. aeruginosa* was present in 16% of patients. A subset of patients did not have organisms with typical CAZ-AVI target resistance mechanisms, including non-carbapenemase-producing Enterobacterales and non-DTR *P. aeruginosa*. To further characterize this population, we evaluated the 108 patients without CRE or carbapenem-resistant *P. aeruginosa*. In this subgroup, the most common infection source was pneumonia (53.7%), followed by urinary tract (15.7%) and skin and soft tissue infections (8.3%). The most frequently isolated organisms were *P. aeruginosa* (31.5%), *K. pneumoniae* (19.4%), and *E. coli* (10.2%). Median duration of CAZ-AVI therapy was 194 h (IQR 117.5–317.6), and only one patient (0.9%) developed treatment-emergent resistance.

### Clinical outcomes

Composite clinical success occurred in 64.8% of patients (Table 4). Individual components included the absence of 30-day all-cause mortality (82.5%), the absence of 30-day microbiological recurrence (91.8%), and resolution of fever and/or leukocytosis within 72 h (80.9%). Infection-related mortality was 12.4%, with a median hospital length of stay of 29.0 days (IQR 13.5–53.8). Adverse events were rare, with CDI and CDI-associated diarrhea being the most common (0.7% each).

**TABLE 4:** Outcomes^*f*^

Parameter^*a*^	(*n* = 613)
Composite clinical success	397 (64.8)
30-day all-cause mortality^*b*^	107 (17.5)
Microbiological recurrence, 30 days from first CAZ/AVI dose	50 (8.2)
Resolution of fever and/or leukocytosis within 72 h	496 (80.9)
Leukocytosis	
No leukocytosis at time of index infection	203 (33.1)
Resolution of elevated white count to within normal limits	165 (26.9)
Reduction in white count from the start of infection but not to within normal limits	124 (20.2)
Infection-related elevation in white count	80 (13.1)
Elevated white count secondary to other causes unrelated to index infection (e.g., CDI and surgery)	40 (6.5)
Fever	
No fever at time of index infection	333 (54.3)
Resolution of fever to within normal limits	221 (36.1)
Infection-related fever continued	28 (4.6)
Reduction in fever from the start of infection but not to within normal limits	19 (3.1)
Fever persistence due to causes unrelated to the index infection (drug fever, etc.)	10 (1.6)
Infection-related mortality^*c*^	76 (12.4)
Adverse drug reaction (ADR)	
CDI	4 (0.7)
CDI-associated diarrhea	4 (0.7)
Neutropenia^*e*^	2 (0.3)
ADR led to drug discontinuation	2 (0.3)
Nephrotoxicity^*d*^	2 (0.3)
Gastrointestinal (nausea, vomiting, and diarrhea)	2 (0.3)
Cardiac (arrhythmias and QTc prolongations)	1 (0.2)
Lactic acidosis	1 (0.2)

^*a*^ Data presented as *n* (%).

^*b*^ 30-day all-cause mortality: Death from any cause within 30 days of index culture collection, regardless of whether the primary cause is attributable to the multidrug-resistant gram-negative infection or other factors, as determined by clinical documentation.

^*c*^ Infection-related mortality: Death within 30 days of index culture primarily due to MDR gram-negative infection, confirmed by clinical signs (e.g., persistent fever, leukocytosis, and positive cultures) or sepsis-related organ failure, excluding causes unrelated to the index infection.

^*d*^ Nephrotoxicity: SCr increase ≥ 0.5 mg/dL and 50% from baseline on two consecutive measurements.

^*e*^ Neutropenia: ANC decrease to <1,500 cells/mm^3^; or 50% decrease in ANC if baseline ANC <1,500 cells/mm^3^ from initiation of antibiotic.

^*f*^ CDI, *C. difficile* infection; CAZ/AVI, ceftazidime/avibactam; ANC, absolute neutrophil count.

Compared to failures, successful patients were younger (median 60 vs 61 years, *P* = 0.034), had lower BMI (median 26 vs 28 kg/m², *P* = 0.008), and lower severity scores (APACHE II: 22 vs 25, *P* < 0.001; SOFA: 5 vs 7, *P* < 0.001). They had higher rates of prior infection with resistant organisms (20.2% vs 12%, *P* = 0.011) but lower rates of heart failure (14.1% vs 20.8%, *P* = 0.032) and class 2 obesity (11.7% vs 7.8%, *P* = 0.024; Table 1). Success patients also had higher rates of appropriate dosing (90.2% vs 83.3%, *P* = 0.014), lower pneumonia prevalence (50.9% vs 63.9%, *P* = 0.002), higher urinary tract infection rates (17.1% vs 11.1%, *P* = 0.046), lower endotracheal aspirate cultures (6.8% vs 13.9%, *P* = 0.004), higher tissue culture rates (6% vs 1.9%, *P* = 0.018), and lower CRE prevalence (33.5% vs 37%, *P* = 0.380), while DTR *P. aeruginosa* was more frequent in failures (17.1% vs 13.6%, *P* = 0.052; Table 2).

### Predictors of clinical success

Multivariable logistic regression identified colonization with resistant organisms (aOR 1.82, 95% CI 1.04–3.20, *P* = 0.037) as a predictor of success (Table 5). Higher SOFA score (aOR 0.87, 95% CI 0.82–0.92, *P* < 0.001) was associated with failure. Higher APACHE II score (aOR 0.97, 95% CI 0.95–1.00, *P* = 0.053) and prior active therapy (aOR 0.67, 95% CI 0.43–1.03, *P* = 0.067) demonstrated trends toward association with failure.

**TABLE 5:** Multivariable logistic regression analysis of predictors of composite clinical success for CAZ-AVI therapy^*a*^^,^^*b*^

Variable	β/aOR	95% CI	*P*-value
Colonization with resistant organisms	1.82	1.04, 3.20	**0.037**
APACHE II score	0.97	0.95, 1.00	0.053
SOFA score	0.87	0.82, 0.92	**<0.001**
Active therapy prior to CAZ/AVI	0.67	0.431, 1.03	0.067

^*a*^ CAZ/AVI, ceftazidime/avibactam; aOR, adjusted odds ratio; CI, confidence interval; APACHE, Acute Physiology and Chronic Health Evaluation; SOFA, sequential organ failure assessment.

^*b*^ Boldface indicates that the *P*-value is significant.

### Timing of CAZ-AVI initiation

Among 603 patients with evaluable timing data, CAZ-AVI initiation within 48 h (*n* = 175) showed a non-significant trend toward higher composite clinical success (70.1% vs 62.1%, *P* = 0.064), driven by improved resolution of fever and/or leukocytosis (89.1% vs 82.4%, *P* = 0.042; Table S1). Given concern for confounding by indication related to receipt of prior active antimicrobial therapy, timing analyses were further evaluated among patients who did not receive active therapy prior to CAZ-AVI initiation. In this subgroup (*n* = 429), CART analysis identified a 42 h threshold; initiation within 42 h (*n* = 131) was associated with higher composite clinical success compared with later initiation (73.3% vs 63.4%, *P* = 0.046) and lower 30-day all-cause mortality (10.7% vs 19.1%, *P* = 0.030; Table 6). Baseline and treatment characteristics for early and late initiation groups within this subgroup are shown in Tables S2 and S3.

**TABLE 6:** Early vs late (42 h): outcomes^*b*^^,^^*c*^^,^^*d*^

Parameter	Total *n* = 429^*a*^	Early (<42 h) *n* = 131	Late (≥42 h) *n* = 298	*P*-value
Composite clinical success	285 (66.4)	96 (73.3)	189 (63.4)	**0.046**
30-day mortality from index culture, all cause	71 (16.6)	14 (10.7)	57 (19.1)	**0.030**
30-day recurrence from CAZ/AVI first dose	41 (9.6)	11 (8.4)	30 (10.1)	0.588
Absence of fever and/or elevated WBC within 72 h of CAZ/AVI	371 (86.5)	116 (88.5)	255 (85.6	0.406

^*a*^ Patients with active therapy prior to CAZ/AVI initiation excluded.

^*b*^ The early vs late variable was calculated based on the hours between index culture and first dose of CAZ/AVI given. In some instances, the difference could not be calculated, and those cases were excluded from any early vs late analyses.

^*c*^ CAZ/AVI, ceftazidime/avibactam.

^*d*^ Boldface indicates that the *P*-value is significant.

In patients with pneumonia as the primary infection source within the no–prior active therapy cohort (*n* = 235), the same 42 h threshold remained discriminatory, with CAZ-AVI initiation within 42 h (*n* = 74) associated with higher composite clinical success (73.0% vs 55.9%, *P* = 0.013) and lower 30-day all-cause mortality (10.8% vs 23.0%, *P* = 0.028; Table S4, S5 and S6). Outcomes were also evaluated by pathogen among patients without prior active therapy, using the same 42 h threshold: in *Pseudomonas aeruginosa* infections (*n* = 253), composite clinical success did not differ significantly (71.8% vs 66.7%, *P* = 0.410; Table S7), whereas in *Klebsiella pneumoniae* infections (*n* = 115), early initiation was associated with lower 30-day all-cause mortality (7.4% vs 25.0%, *P* = 0.049; Table S8).

## DISCUSSION

This multicenter retrospective study across 22 U.S. medical centers provides real-world evidence on CAZ-AVI effectiveness for MDR gram-negative infections, emphasizing the timing of therapy initiation in a diverse cohort with complex infections. CAZ-AVI demonstrated robust *in vitro* activity against *P. aeruginosa* and CRE in our cohort (96.8% susceptibility for *P. aeruginosa*, including 97.1% for carbapenem-resistant isolates, and 87.4% for CRE), aligning with a prior systematic review reporting high success rates in carbapenem-resistant infections (14). The large cohort and varied infection profile, including pneumonia, bloodstream infection, and urinary tract infection, broaden insights compared with smaller, pathogen- or site-specific studies (11–13). Novel findings, including a clinically relevant timing threshold identified in a confounding-mitigated cohort and differential susceptibility patterns, provide important guidance for managing MDR infections in settings with diagnostic delays.

Unlike prior studies highlighting a 48 h threshold for early antibiotic therapy (7–9, 11), we observed no statistically significant difference in composite clinical success at this cutoff in the overall cohort. Diagnostic delays, with susceptibility confirmation often exceeding 60 h, likely limited the feasibility of earlier targeted therapy. The observed inverse association between prior active therapy and clinical success likely reflects confounding by indication, which informed our decision to focus timing analyses on patients who did not receive active antimicrobial therapy prior to CAZ-AVI initiation. In this subgroup, CART analysis identified a 42 h threshold, with earlier initiation associated with higher clinical success and lower 30-day mortality. This same threshold remained discriminatory in patients with pneumonia, a population in whom early effective therapy is particularly critical. These findings underscore the interplay among diagnostic feasibility, infection severity, and timing of therapy, and highlight the potential value of rapid diagnostics in severe gram-negative infections (25).

The reliance of the composite outcome on both clinical and laboratory criteria may have reduced sensitivity to timing effects, as resolution of fever and/or leukocytosis occurred in only a subset of patients and may be influenced by causes unrelated to the index infection (26, 27). Mortality outcomes were not uniformly improved across the overall cohort; however, among patients who did not receive active antimicrobial therapy prior to CAZ-AVI initiation, earlier therapy within 42 h was associated with lower 30-day all-cause mortality, particularly in those with pneumonia. Given the high severity of illness (median APACHE II 23, SOFA 5) and the predominance of pneumonia, the absence of a broader mortality signal in the full cohort is not unexpected, as severe respiratory infections are consistently associated with worse outcomes regardless of antimicrobial choice or timing (28–31).

Microbiologically, CAZ-AVI demonstrated strong activity against *P. aeruginosa* and CRE, with comparatively lower susceptibility observed among *K. pneumoniae*, consistent with known resistance mechanisms including KPC variant mutations, MBL production, and efflux-mediated resistance (32–34). In organism-specific analyses restricted to patients without prior active therapy, early CAZ-AVI initiation within 42 h was associated with lower 30-day all-cause mortality in *K. pneumoniae* infections, whereas no significant differences in composite clinical success or mortality were observed in *P. aeruginosa* infections, suggesting pathogen-specific variability in the clinical impact of early therapy. Emergence of CAZ-AVI resistance during or after treatment occurred infrequently and without consistent clinical predictors, reflecting ongoing global challenges with resistance evolution and underscoring the importance of routine susceptibility monitoring (14, 15, 17, 35). Notably, emergent resistance occurred in 12.6% of patients who underwent repeat susceptibility testing (4.1% overall), highlighting the importance of contextualizing resistance rates based on testing frequency. Additionally, a subset of patients received CAZ-AVI in the absence of typical target resistance mechanisms, including non-carbapenemase-producing Enterobacterales and non-DTR *P. aeruginosa*. Outcomes in this group, along with the low rate of emergent resistance, suggest that empiric or pre-emptive use may occur in high-risk settings, but underscore the importance of antimicrobial stewardship and early de-escalation once susceptibility data are available. Overall, these findings reinforce that microbiologic factors, host severity, and treatment context collectively influence outcomes with CAZ-AVI.

Polymicrobial infections, though less common, may have complicated outcomes through pathogen–pathogen interactions (36, 37). Compared with other novel β-lactam/β-lactamase inhibitors, CAZ-AVI retains an advantage against CRE but showed variable efficacy against *K. pneumoniae*, emphasizing the need for individualized therapy guided by susceptibility (38–40). Meropenem–vaborbactam may serve as an alternative in KPC-producing isolates but lacks broader anti*-P. aeruginosa* coverage.

Appropriate renal-based dosing (achieved in 87.8%) remained critical, while combination therapy (17% of cases) offered limited benefit, consistent with prior real-world studies (41–43). High rates of infectious disease consultation (94.3%) reflect the complexity of decision-making and likely contributed to optimized use, though rationales varied (e.g., no active alternatives in 40.3% and consolidation in 22.8%). Safety outcomes were favorable, with very low rates of CDI and CDI-associated diarrhea (each 0.7%) and nephrotoxicity (0.3%), supporting the tolerability advantage of CAZ-AVI over older agents (44–46).

Limitations include the retrospective design, absence of a comparator arm, and non-standardized resistance testing. Although inverse probability weighting was applied in the full cohort, residual confounding, particularly confounding by indication, remains a concern in observational timing analyses. Focused analyses among patients without prior active antimicrobial therapy were performed to improve the internal validity of the timing assessment. The large, geographically diverse cohort enhances generalizability across U.S. hospitals, although applicability to rural settings may be limited (47, 48). CART analyses, including those conducted in this study, are intended to be exploratory in nature and should not solely guide clinical decision-making (49).

Subgroup analyses confirmed worse outcomes in pneumonia compared with urinary tract infections, consistent with known challenges related to drug penetration into lung tissue, high illness severity, and ventilator-associated complications (50–52). Ceftazidime-avibactam exhibits variable epithelial lining fluid (ELF) penetration, with reported ELF-to-plasma ratios that may be suboptimal in critically ill patients, particularly in the setting of altered pulmonary permeability and augmented renal clearance, which may limit target attainment in severe pneumonia. Within patients who did not receive active antimicrobial therapy prior to CAZ-AVI initiation, the 42 h timing threshold identified by CART analysis remained discriminatory in pneumonia, underscoring the importance of early effective therapy in severe respiratory infections, and the potential impact of diagnostic delays.

Clinically, these findings support initiating CAZ-AVI as early as feasible among patients without prior active antimicrobial therapy, ideally within 42 h of index culture collection, including those with pneumonia. Integration of rapid diagnostic tools, optimization of renal-based dosing, close monitoring for *Klebsiella pneumoniae* resistance, and early involvement of infectious disease specialists remain critical components of care. Future prospective studies are needed to validate timing thresholds in diverse clinical settings, while molecular investigations should further elucidate resistance mechanisms to inform antimicrobial stewardship strategies.

## SUPPLEMENTAL MATERIAL

Supplemental tables aac.00268-26-s0001.docx Tables S1 to S8.

ASM does not own the copyrights to Supplemental Material that may be linked to, or accessed through, an article. The authors have granted ASM a non-exclusive, world-wide license to publish the Supplemental Material files. Please contact the corresponding author directly for reuse.
