# Supplementary material for: Ceftazidime-avibactam for multidrug-resistant gram-negative infections: outcomes and timing of initiation across 22 U.S. medical centers
Source: Antimicrob Agents Chemother. 2026 May 15;70(6):e00268-26. doi: 10.1128/aac.00268-26 (PMC13231909; doi:10.1128/aac.00268-26)
Supplement: Supplemental tables — Tables S1 to S8. [file aac.00268-26-s0001.docx]

**Supplemental Table 1.** Early vs. Late: Comparing Rates of Success Between Definitions (*n*=603)

| **Parameter** | **Early (≤48 hrs)**  ***n*=175** | **Late (>48 hrs)**  ***n*=428** | **p-value** |
| --- | --- | --- | --- |
| Clinical success | 122 (70.1) | 266 (62.1) | 0.064 |
| 30-day mortality from index culture, all cause | 26 (14.9) | 81 (18.9) | 0.235 |
| 30-day recurrence from CAZ/AVI first dose | 15 (8.6) | 35 (8.2) | 0.874 |
| Absence of fever and elevated WBC within 72 hrs of CAZ/AVI | 155 (89.1) | 352 (82.4) | **0.042** |

*Note:* The early vs. late variable was calculated based on the hours between index culture and first dose of CAZ/AVI given. In some instances, the difference could not be calculated, and those cases were excluded from any Early vs. Late analyses.

Abbreviations: CAZ/AVI, ceftazidime/avibactam

**Supplemental Table 2.** Baseline Characteristics, Early vs. Late (42-Hour), for Patients Not Receiving Active Antimicrobial Therapy Prior to CAZ-AVI

| **Parameter** | **Total**  ***n*=429** | **Early (<42)**  ***n*=131** | **Late (≥42)**  ***n*=298** | **p-value** |
| --- | --- | --- | --- | --- |
| **Age** (years), median (IQR) | 59 (49, 69) | 58 (44, 69) | 60 (50, 69) | 0.064 |
| **Male** | 267 (62.2) | 88 (67.2) | 179 (60.1) | 0.162 |
| **Race**  Caucasian  African American  Hispanic  Asian  Other | 197 (45.9)  141 (32.9)  44 (10.3)  8 (1.9)  38 (8.9) | 58 (44.3)  49 (37.4)  12 (9.2)  2 (1.5)  9 (6.9) | 139 (46.6)  92 (30.9)  32 (10.7)  6 (2)  29 (9.7) | 0.650  0.185  0.620  0.731  0.337 |
| **BMI (kg/m^2^),** median (IQR) | 26.6 (22.5, 32.4) | 25.2 (21.3, 31.5) | 27.0 (23.1, 32.8) | **0.029** |
| **Obese** (BMI ≥30 kg/m^2^)  Class 1  Class 2  Class 3 | 69 (16.1)  40 (9.3)  34 (7.9) | 16 (12.2)  12 (9.2)  6 (4.6) | 53 (17.8)  28 (9.4)  28 (9.4) | 0.148  0.938  0.089 |
| **Admitted from**  Home  Nursing home/Skilled Nursing Facility  Transfer from outside hospital  Referral from clinic  Long term acute care (LTAC)  Inpatient rehab facility  Unhoused | 206 (48)  115 (26.8)  67 (15.6)  20 (4.7)  14 (3.3)  6 (1.4)  1 (0.2) | 61 (46.6)  42 (32.1)  12 (9.2)  10 (7.6)  4 (3.1)  2 (1.5)  0 (0) | 145 (48.7)  73 (24.5)  55 (18.5)  10 (3.4)  10 (3.4)  4 (1.3)  1 (0.3) | 0.689  0.103  **0.015**  0.053  0.871  0.881  0.507 |
| **Severity scores**  APACHE II score  SOFA score  Charlson Comorbidity Index (CCI) | 23 (16, 28)  6 (3, 8)  2 (1, 4) | 23 (16, 29)  6 (3, 8)  2 (1, 4) | 23 (16, 28)  6 (3, 8)  2 (1, 4) | 0.719  0.598  0.776 |
| **Immunosuppression**  Cytotoxic chemotherapy in preceding 90 days  Neutropenia (ANC or WBC < 500)  High-dose corticosteroids  Solid organ transplant in preceding 90 days  Splenectomy (functional or surgical)  Bone marrow transplant in preceding 90 days  AIDS or AIDS defining illness | 23 (5.4)  21 (4.9)  17 (4)  13 (3)  3 (0.7)  3 (0.7)  2 (0.5) | 5 (3.8)  4 (3.1)  6 (4.6)  4 (3.1)  2 (1.5)  1 (0.8)  0 (0) | 18 (6)  17 (5.7)  11 (3.7)  9 (3)  1 (0.3)  2 (0.7)  2 (0.7) | 0.346  0.241  0.664  0.985  0.173  0.916  0.347 |
| **MDR risk factors**  Hospitalization ≥48 hours in 90 days before IC  Antimicrobials ≥24 hours in 90 days before IC  Prior infection with resistant organisms  Admitted from NH or extended care facility  Colonization with resistant organisms  Surgery in 30 days before index culture  Chronic dialysis in 30 days before index culture  Home wound care  Home infusion (includes antibiotics) | 309 (72)  283 (66)  135 (31.5)  123 (28.7)  81 (18.9)  46 (10.7)  30 (7)  21 (4.9)  14 (3.3) | 95 (72.5)  84 (64.1)  55 (42)  40 (30.5)  35 (26.7)  9 (6.9)  6 (4.6)  11 (8.4)  3 (2.3) | 214 (71.8)  199 (66.8)  80 (26.8)  83 (27.9)  46 (15.4)  37 (12.4)  24 (8.1)  10 (3.4)  11 (3.7) | 0.881  0.593  **0.002**  0.572  **0.006**  0.087  0.194  **0.026**  0.542 |
| **Comorbid conditions**  Diabetes  Heart failure  Moderate to severe CKD  Cerebrovascular disease  COPD  Peripheral vascular disease  COVID-19  Acute kidney injury  Myocardial infarction  Asthma  Chronic dialysis  Hemiplegia  Dementia  Connective tissue disease  Tumor with metastasis  Tumor without metastasis  Cystic fibrosis  Moderate to severe liver disease  Leukemia  IV drug use  *Clostridium difficile* associated diarrhea  Peptic ulcer disease  HIV  Mild liver disease  Lymphoma  AIDS | 182 (42.4)  71 (16.6)  68 (15.9)  67 (15.6)  64 (14.9)  58 (13.5)  51 (11.9)  48 (11.2)  39 (9.1)  35 (8.2)  31 (7.2)  23 (5.4)  22 (5.1)  18 (4.2)  18 (4.2)  15 (3.5)  14 (3.3)  14 (3.3)  11 (2.6)  7 (1.6)  6 (1.4)  6 (1.4)  5 (1.2)  5 (1.2)  3 (0.7)  2 (0.5) | 62 (47.3)  18 (13.7)  22 (16.8)  18 (13.7)  18 (13.7)  17 (13.0)  6 (4.6)  11 (8.4)  9 (6.9)  9 (6.9)  7 (5.3)  10 (7.6)  8 (6.1)  6 (4.6)  4 (3.1)  4 (3.1)  9 (6.9)  2 (1.5)  3 (2.3)  5 (3.8)  3 (2.3)  2 (1.5)  1 (0.8)  1 (0.8)  2 (1.5)  0 (0.0) | 120 (40.3)  53 (17.8)  46 (15.4)  49 (16.4)  46 (15.4)  41 (13.8)  45 (15.1)  37 (12.4)  30 (10.1)  26 (8.7)  24 (8.1)  13 (4.4)  14 (4.7)  12 (4.0)  14 (4.7)  11 (3.7)  5 (1.7)  12 (4.0)  8 (2.7)  2 (0.7)  3 (1.0)  4 (1.3)  4 (1.3)  4 (1.3)  1 (0.3)  2 (0.7) | 0.173  0.299  0.723  0.478  0.650  0.827  **0.002**  0.224  0.289  0.518  0.318  0.166  0.542  0.792  0.434  0.740  **0.005**  0.180  0.812  **0.018**  0.297  0.881  0.607  0.607  0.173  0.347 |

**Abbreviations:** IQR, interquartile range; BMI, body mass index; NH, nursing home; APACHE, Acute Physiology and Chronic Health Evaluation; SOFA, sequential organ failure assessment; ANC, absolute neutrophil count; WBC, white blood cell; AIDS, acquired immunodeficiency syndrome; COPD, chronic obstructive pulmonary disease; CKD, chronic kidney disease; HIV, human immunodeficiency virus; IV, intravenous

**Supplemental Table 3.** Clinical course and treatment characteristics, Early vs. Late (42-hour), for Patients Not Receiving Active Antimicrobial Therapy Prior to CAZ-AVI

| **Parameter** | **Total**  ***n*=429** | **Early (<42)**  ***n*=131** | **Late (≥42)**  ***n*=298** | **p-val** |
| --- | --- | --- | --- | --- |
| **Culture source**  Sputum  Blood  Urine  Endotracheal aspirate  Bronchoalveolar lavage  Wound  Fluid  Tissue  Bone  Other | 164 (38.2)  77 (17.9)  62 (14.5)  39 (9.1)  32 (7.5)  29 (6.8)  29 (6.8)  17 (4.0)  5 (1.2)  11 (2.6) | 59 (45.0)  25 (19.1)  21 (16.0)  9 (6.9)  8 (6.1)  4 (3.1)  5 (3.8)  5 (3.8)  1 (0.8)  3 (2.3) | 105 (35.2)  52 (17.4)  41 (13.8)  30 (10.1)  24 (8.1)  25 (8.4)  24 (8.1)  12 (4.0)  4 (1.3)  8 (2.7) | 0.054  0.685  0.538  0.289  0.480  **0.043**  0.107  0.918  0.607  0.812 |
| **Organisms targeted by CAZ/AVI^a^**  *Pseudomonas aeruginosa*  *Klebsiella pneumoniae*  *Escherichia coli*  *Enterobacter cloacae*  *Serratia marcescens*  *Enterobacter aerogenes*  *Proteus mirabilis*  *Citrobacter freundii*  *Providencia stuartii*  Other *Citrobacter*  *Klebsiella oxytoca*  *Morganella morganii* | 253 (59.0)  115 (26.8)  29 (6.8)  25 (5.8)  12 (2.8)  11 (2.6)  8 (1.9)  6 (1.4)  2 (0.5)  2 (0.5)  2 (0.5)  2 (0.5) | 85 (64.9)  27 (20.6)  9 (6.9)  9 (6.9)  3 (2.3)  2 (1.5)  6 (4.6)  1 (0.8)  2 (1.5)  1 (0.8)  0 (0.0)  0 (0.0) | 168 (56.4)  88 (29.5)  20 (6.7)  16 (5.4)  9 (3.0)  9 (3.0)  2 (0.7)  5 (1.7)  0 (0.0)  1 (0.3)  2 (0.7)  2 (0.7) | 0.099  0.055  0.952  0.541  0.673  0.367  **0.006**  0.458  **0.033**  0.549  0.347  0.347 |
| **Polymicrobial infection** | 210 (49.1) | 59 (45.0) | 151 (50.8) | 0.268 |
| **Resistance phenotypes**  CRE  DTR, *P. aeruginosa* as primary organism  MDR, *P. aeruginosa* as primary organism | 163 (38.0)  70 (16.3)  29 (6.8) | 33 (25.2)  16 (12.2)  14 (10.7) | 130 (43.6)  54 (18.1)  15 (5.0) | **<0.001**  0.127  **0.032** |
| **Combination therapies**  Inhaled tobramycin  Inhaled colistin  Inhaled amikacin  Aztreonam  Tobramycin  Amikacin  Colistin  Gentamicin  Meropenem  Tigecycline  Ciprofloxacin, IV  Polymyxin  Cefepime  Levofloxacin, IV  Eravacycline  Trimethoprim-Sulfamethoxazole | 26 (6.1)  5 (1.2)  1 (0.2)  13 (3.0)  13 (3.0)  8 (1.9)  3 (0.7)  3 (0.7)  3 (0.7)  3 (0.7)  2 (0.5)  2 (0.5)  1 (0.2)  1 (0.2)  1 (0.2)  1 (0.2) | 6 (4.6)  2 (1.5)  0 (0.0)  5 (3.8)  4 (3.1)  3 (2.3)  1 (0.8)  0 (0.0)  1 (0.8)  1 (0.8)  0 (0.0)  1 (0.8)  1 (0.8)  1 (0.8)  0 (0.0)  0 (0.0) | 20 (6.7)  3 (1.0)  1 (0.3)  8 (2.7)  9 (3.0)  5 (1.7)  2 (0.7)  3 (1.0)  2 (0.7)  2 (0.7)  2 (0.7)  1 (0.3)  0 (0.0)  0 (0.0)  1 (0.3)  1 (0.3) | 0.394  0.644  0.507  0.529  0.985  0.666  0.916  0.249  0.916  0.916  0.347  0.549  0.131  0.131  0.507  0.507 |
| **ICU admissions**  1 admission  2 admissions  3 admissions | 212 (49.4)  58 (13.5)  28 (6.5) | 67 (51.1)  16 (12.2)  7 (5.3) | 145 (48.7)  42 (14.1)  21 (7.0) | 0.635  0.600  0.511 |
| **ID consult** | 403 (93.9) | 119 (90.8) | 284 (95.3) | 0.074 |
| **Rationale for CAZ/AVI use**  No other active agent for infection  Double coverage for suspected CRE/C-R PsA  Consolidation of regimen  Antibiotic shortage  Other | 182 (42.4)  108 (25.2)  90 (21.0)  37 (8.6)  59 (13.8) | 41 (31.3)  48 (36.6)  32 (24.4)  7 (5.3)  18 (13.7) | 141 (47.3)  60 (20.1)  58 (19.5)  30 (10.1)  41 (13.8) | **0.002**  **<0.001**  0.245  0.108  0.996 |
| **CAZ/AVI treatment**  0.94 g every 12 hours  0.94 g every 24 hours  0.94 g every 48 hours  1.25 g every 8 hours  1.25 g every 12 hours  1.25 g every 24 hours  2.5 g every 8 hours  2.5 g every 12 hours | 12 (2.8)  22 (5.1)  9 (2.1)  75 (17.5)  6 (1.4)  3 (0.7)  293 (68.3)  1 (0.2) | 5 (3.8)  5 (3.8)  0 (0.0)  25 (19.1)  1 (0.8)  2 (1.5)  92 (70.2)  0 (0.0) | 7 (2.3)  17 (5.7)  9 (3.0)  50 (16.8)  5 (1.7)  1 (0.3)  201 (67.4)  1 (0.3) | 0.396  0.415  **0.044**  0.563  0.458  0.173  0.569  0.507 |
| **Appropriate CAZ/AVI dose based on CrCl** | 382 (89.0) | 121 (92.4) | 261 (87.6) | 0.144 |
| **Hospital length of stay** (days) | 28.0 (13.8, 52.5) | 16.0 (8.6, 39.0) | 31.7 (16.4, 57.0) | **<0.001** |
| **Discharge disposition**  NH, Skilled nursing facility, Long-term acute care  Home  Morgue  Hospice  Rehabilitation center | 158 (37.0)  136 (31.7)  78 (18.3)  31 (7.3)  24 (5.6) | 48 (36.9)  48 (36.6)  16 (12.3)  11 (8.5)  7 (5.4) | 110 (37.0)  88 (29.5)  62 (20.9)  20 (6.7)  17 (5.7) | 0.982  0.145  **0.035**  0.527  0.889 |

^a^ The total number of identified organisms exceeds the total study population due to polymicrobial infections containing multiple organisms. Percentages were determined using the total number of isolates as the denominator.

^b^Percentages were calculated using the number of applicable isolates as the denominator.

^c^Associated p-val refers to the distribution of MDR PsA and DTR PsA between Early and Late groups.

Abbreviations: UTI, urinary tract infection; IV, intravenous; CNS, central nervous system; CAZ/AVI, ceftazidime/avibactam; MRSA, methicillin-susceptible *Staphylococcus aureus*; MSSA, methicillin-susceptible *Staphylococcus aureus*; CoNS, coagulase negative *Staphylococcus aureus*; CRE, carbapenem-resistant Enterobacterales; DTR, difficult-to-treat; MDR, multidrug-resistant; TMP-SMX, trimethoprim-sulfamethoxazole; ICU, intensive care unit; ID, infectious diseases; PO, oral; NH, nursing home

**Supplemental Table 4.** Baseline Characteristics Among Patients With Pneumonia as the Primary Infection Source for Patients Not Receiving Active Antimicrobial Therapy Prior to CAZ-AVI

| **Parameter** | **Total**  ***n*=235** | **Early (<42)**  ***n*=74** | **Late (≥42)**  ***n*=161** | **p-value** |
| --- | --- | --- | --- | --- |
| **Age** (years), median (IQR) | 60.0 (47.0, 69.0) | 60.0 (44.0, 69.0) | 60.0 (49.0, 70.0) | 0.364 |
| **Male** | 149 (63.4) | 53 (71.6) | 96 (59.6) | 0.076 |
| **Race**  Caucasian  African American  Hispanic  Asian  Other | 114 (48.5)  75 (31.9)  21 (8.9)  2 (0.9)  23 (9.8) | 39 (52.7)  26 (35.1)  5 (6.8)  0 (0.0)  4 (5.4) | 75 (46.6)  49 (30.4)  16 (9.9)  2 (1.2)  19 (11.8) | 0.383  0.473  0.427  0.336  0.125 |
| **BMI (kg/m^2^),** median (IQR) | 26.0 (22.4, 32.3) | 23.7 (20.5, 28.6) | 26.8 (23.7, 34.1) | **<0.001** |
| **Obese** (BMI ≥30 kg/m^2^)  Class 1  Class 2  Class 3 | 34 (14.5)  28 (11.9)  14 (6.0) | 7 (9.5)  7 (9.5)  1 (1.4) | 27 (16.8)  21 (13.0)  13 (8.1) | 0.139  0.431  **0.043** |
| **Admitted from**  Home  Nursing home/Skilled Nursing Facility  Transfer from outside hospital  Referral from clinic  Long term acute care (LTAC)  Inpatient rehab facility  Unhoused | 97 (41.3)  80 (34.0)  40 (17.0)  8 (3.4)  7 (3.0)  2 (0.9)  1 (0.4) | 27 (36.5)  33 (44.6)  6 (8.1)  5 (6.8)  3 (4.1)  0 (0.0)  0 (0.0) | 70 (43.5)  47 (29.2)  34 (21.1)  3 (1.9)  4 (2.5)  2 (1.2)  1 (0.6) | 0.312  **0.021**  **0.014**  0.055  0.511  0.336  0.497 |
| **Severity scores**  APACHE II score  SOFA score  Charlson Comorbidity Index (CCI) | 24.0 (18.0, 29.0)  6.0 (3.0, 8.0)  2.0 (1.0, 3.0) | 24.0 (18.0, 29.5)  6.0 (3.0, 8.3)  2.0 (1.0, 3.0) | 24.0 (18.0, 29.0)  6.0 (4.0, 8.0)  2.0 (1.0, 3.0) | 0.980  0.690  0.939 |
| **Immunosuppression**  Neutropenia (ANC or WBC < 500)  High-dose corticosteroids  Solid organ transplant in preceding 90 days  Cytotoxic chemotherapy in preceding 90 days  Splenectomy (functional or surgical) | 12 (5.1)  10 (4.3)  7 (3.0)  6 (2.6)  1 (0.4) | 2 (2.7)  4 (5.4)  4 (5.4)  1 (1.4)  1 (1.4) | 10 (6.2)  6 (3.7)  3 (1.9)  5 (3.1)  0 (0.0) | 0.256  0.554  0.138  0.428  0.139 |
| **MDR risk factors**  Hospitalization ≥48 hours in 90 days before IC  Antimicrobials ≥24 hours in 90 days before IC  Admitted from NH or extended care facility  Prior infection with resistant organisms  Colonization with resistant organisms  Surgery in 30 days before index culture  Chronic dialysis in 30 days before index culture  Home wound care  Home infusion (includes antibiotics) | 169 (71.9)  155 (66.0)  85 (36.2)  75 (31.9)  47 (20.0)  22 (9.4)  12 (5.1)  12 (5.1)  5 (2.1) | 53 (71.6)  46 (62.2)  31 (41.9)  30 (40.5)  20 (27.0)  5 (6.8)  2 (2.7)  6 (8.1)  2 (2.7) | 116 (72.0)  109 (67.7)  54 (33.5)  45 (28.0)  27 (16.8)  17 (10.6)  10 (6.2)  6 (3.7)  3 (1.9) | 0.946  0.405  0.216  0.054  0.068  0.353  0.256  0.156  0.679 |
| **Comorbid conditions**  Diabetes  COPD  COVID-19  Cerebrovascular disease  Heart failure  Moderate to severe CKD  Peripheral vascular disease  Acute kidney injury  Myocardial infarction  Asthma  Dementia  Connective tissue disease  Cystic fibrosis  Chronic dialysis  Hemiplegia  Tumor with metastasis  Tumor without metastasis  IV drug use  Peptic ulcer disease  Moderate to severe liver disease  Leukemia  *Clostridium difficile* associated diarrhea  HIV  Lymphoma | 95 (40.4)  45 (19.1)  43 (18.3)  41 (17.4)  38 (16.2)  33 (14.0)  23 (9.8)  23 (9.8)  23 (9.8)  21 (8.9)  15 (6.4)  14 (6.0)  13 (5.5)  11 (4.7)  9 (3.8)  6 (2.6)  6 (2.6)  5 (2.1)  4 (1.7)  4 (1.7)  3 (1.3)  1 (0.4)  1 (0.4)  1 (0.4) | 35 (47.3)  13 (17.6)  6 (8.1)  12 (16.2)  9 (12.2)  12 (16.2)  6 (8.1)  6 (8.1)  5 (6.8)  6 (8.1)  6 (8.1)  5 (6.8)  9 (12.2)  2 (2.7)  3 (4.1)  2 (2.7)  1 (1.4)  3 (4.1)  0 (0.0)  0 (0.0)  2 (2.7)  0 (0.0)  1 (1.4)  1 (1.4) | 60 (37.3)  32 (19.9)  37 (23.0)  29 (18.0)  29 (18.0)  21 (13.0)  17 (10.6)  17 (10.6)  18 (11.2)  15 (9.3)  9 (5.6)  9 (5.6)  4 (2.5)  9 (5.6)  6 (3.7)  4 (2.5)  5 (3.1)  2 (1.2)  4 (2.5)  4 (2.5)  1 (0.6)  1 (0.6)  0 (0.0)  0 (0.0) | 0.146  0.676  **0.006**  0.736  0.258  0.51  0.557  0.557  0.289  0.763  0.463  0.726  **0.003**  0.330  0.903  0.922  0.428  0.165  0.171  0.171  0.187  0.497  0.139  0.139 |

**Abbreviations:** IQR, interquartile range; BMI, body mass index; NH, nursing home; APACHE, Acute Physiology and Chronic Health Evaluation; SOFA, sequential organ failure assessment; ANC, absolute neutrophil count; WBC, white blood cell; IC, index culture; COPD, chronic obstructive pulmonary disease; CKD, chronic kidney disease; HIV, human immunodeficiency virus; IV, intravenous

**Supplemental Table 5.** Clinical Course and Treatment Characteristics Among Patients With Pneumonia as the Primary Infection Source for Patients Not Receiving Active Antimicrobial Therapy Prior to CAZ-AVI

| **Parameter** | **Total**  ***n*=235** | **Early (<42)**  ***n*=74** | **Late (≥42)**  ***n*=161** | **p-value** |
| --- | --- | --- | --- | --- |
| **Culture source**  Sputum  Endotracheal aspirate  Bronchoalveolar lavage  Blood  Fluid  Tissue  Other | 155 (66.0)  36 (15.3)  29 (12.3)  10 (4.3)  4 (1.7)  4 (1.7)  6 (2.6) | 54 (73.0)  9 (12.2)  7 (9.5)  3 (4.1)  0 (0.0)  2 (2.7)  1 (1.4) | 101 (62.7)  27 (16.8)  22 (13.7)  7 (4.3)  4 (2.5)  2 (1.2)  5 (3.1) | 0.124  0.362  0.363  0.917  0.171  0.421  0.428 |
| **Organisms targeted by CAZ/AVI^a^**  *Pseudomonas aeruginosa*  *Klebsiella pneumoniae*  *Proteus mirabilis*  *Escherichia coli*  *Serratia marcescens*  *Enterobacter cloacae*  *Providencia stuartii*  *Enterobacter aerogenes*  Other *Citrobacter*  *Citrobacter freundii*  *Klebsiella oxytoca* | 173 (73.6)  46 (19.6)  6 (2.6)  6 (2.6)  9 (3.8)  5 (2.1)  1 (0.4)  4 (1.7)  2 (0.9)  3 (1.3)  1 (0.4) | 60 (81.1)  10 (13.5)  4 (5.4)  3 (4.1)  1 (1.4)  1 (1.4)  1 (1.4)  0 (0.0)  1 (1.4)  0 (0.0)  0 (0.0) | 113 (70.2)  36 (22.4)  2 (1.2)  3 (1.9)  8 (5.0)  4 (2.5)  0 (0.0)  4 (2.5)  1 (0.6)  3 (1.9)  1 (0.6) | 0.078  0.112  0.060  0.323  0.180  0.576  0.139  0.171  0.571  0.237  0.497 |
| **Polymicrobial infection** | 121 (51.7) | 40 (54.1) | 81 (50.6) | 0.625 |
| **Resistance phenotypes**  CRE  DTR, *P. aeruginosa* as primary organism  MDR, *P. aeruginosa* as primary organism | 58 (24.7)  50 (21.3)  16 (6.8) | 8 (10.8)  13 (17.6)  7 (9.5) | 50 (31.1)  37 (23.0)  9 (5.6) | **<0.001**  0.346  0.274 |
| **Combination therapies**  Inhaled tobramycin  Inhaled colistin  Inhaled amikacin  Tobramycin  Aztreonam  Amikacin  Colistin  Polymyxin  Gentamicin  Meropenem  Cefepime | 26 (11.1)  5 (2.1)  1 (0.4)  11 (4.7)  3 (1.3)  3 (1.3)  2 (0.9)  1 (0.4)  1 (0.4)  1 (0.4)  1 (0.4) | 6 (8.1)  2 (2.7)  0 (0.0)  3 (4.1)  1 (1.4)  0 (0.0)  0 (0.0)  0 (0.0)  0 (0.0)  1 (1.4)  1 (1.4) | 20 (12.4)  3 (1.9)  1 (0.6)  8 (5.0)  2 (1.2)  3 (1.9)  2 (1.2)  1 (0.6)  1 (0.6)  0 (0.0)  0 (0.0) | 0.327  0.679  0.497  0.758  0.945  0.237  0.336  0.497  0.497  0.139  0.139 |
| **ICU admissions**  1 admission  2 admissions  3 admissions | 148 (63.0)  35 (14.9)  13 (5.5) | 45 (60.8)  12 (16.2)  2 (2.7) | 103 (64.0)  23 (14.3)  11 (6.8) | 0.641  0.699  0.198 |
| **ID consult** | 215 (91.5) | 65 (87.8) | 150 (93.2) | 0.174 |
| **Rationale for CAZ/AVI use**  No other active agent for infection  Double coverage for suspected CRE/C-R PsA  Consolidation of regimen  Antibiotic shortage  Other | 97 (41.3)  64 (27.2)  40 (17.0)  22 (9.4)  35 (14.9) | 23 (31.1)  32 (43.2)  14 (18.9)  3 (4.1)  9 (12.2) | 74 (46.0)  32 (19.9)  26 (16.1)  19 (11.8)  26 (16.1) | **0.031**  **<0.001**  0.600  0.058  0.425 |
| **CAZ/AVI treatment**  0.94 g every 12 hours  0.94 g every 24 hours  0.94 g every 48 hours  1.25 g every 8 hours  1.25 g every 12 hours  1.25 g every 24 hours  2.5 g every 8 hours  2.5 g every 12 hours | 6 (2.6)  9 (3.8)  5 (2.1)  33 (14.0)  5 (2.1)  1 (0.4)  170 (72.3)  1 (0.4) | 3 (4.1)  1 (1.4)  0 (0.0)  13 (17.6)  0 (0.0)  0 (0.0)  56 (75.7)  0 (0.0) | 3 (1.9)  8 (5.0)  5 (3.1)  20 (12.4)  5 (3.1)  1 (0.6)  114 (70.8)  1 (0.6) | 0.323  0.180  0.125  0.292  0.125  0.497  0.438  0.497 |
| **Appropriate CAZ/AVI dose based on CrCl** | 212 (90.2) | 69 (93.2) | 143 (88.8) | 0.289 |
| **Hospital length of stay** (days) | 30.8 (15.7, 58.0) | 17.2 (9.1, 39.0) | 35.4 (18.9, 67.5) | **<0.001** |
| **Composite Clinical Success**  Absence of fever and leukocytosis 72 hrs after first CAZ/AVI dose  30-day mortality from index culture, all cause  30-day recurrence from CAZ/AVI first dose | 144 (61.3)  195 (83.0)  45 (19.1)  27 (11.5) | 54 (73.0)  64 (86.5)  8 (10.8)  7 (9.5) | 90 (55.9)  131 (81.4)  37 (23.0)  20 (12.4) | **0.013**  0.332  **0.028**  0.508 |
| **Discharge disposition**  NH, Skilled nursing facility, Long-term acute care  Morgue  Home  Hospice  Rehabilitation center | 111 (47.4)  49 (20.9)  48 (20.4)  17 (7.3)  9 (3.8) | 36 (48.6)  11 (14.9)  19 (25.7)  6 (8.1)  2 (2.7) | 75 (46.9)  38 (23.8)  29 (18.0)  11 (6.9)  7 (4.4) | 0.801  0.120  0.176  0.735  0.536 |

^a^ The total number of identified organisms exceeds the total study population due to polymicrobial infections containing multiple organisms. Percentages were determined using the total number of isolates as the denominator.

Abbreviations: CAZ/AVI, ceftazidime/avibactam, CRE, carbapenem-resistant Enterobacterales; DTR, difficult-to-treat resistance; MDR, multi-drug resistance; C-R, carbapenem-resistant; PsA, *Pseudomonas aeruginosa;* CrCl, creatinine clearance

**Supplemental Table 6.** Outcomes for Subpopulations With Respiratory Tract/Pneumonia as the Infection Source Not Receiving Active Antimicrobial Therapy Prior to CAZ-AVI

|  | **Total**  ***n*=235** | **Early (<42)**  ***n*=74** | **Late (≥42)**  ***n*=161** | **p-val** |
| --- | --- | --- | --- | --- |
| **Composite Clinical Success** | 144 (61.3) | 54 (73.0) | 90 (55.9) | **0.013** |
| Absence of fever and leukocytosis 72 hrs after first CAZ/AVI dose | 195 (83.0) | 64 (86.5) | 131 (81.4) | 0.332 |
| 30-day mortality from index culture, all cause | 45 (19.1) | 8 (10.8) | 37 (23.0) | **0.028** |
| 30-day recurrence from CAZ/AVI first dose | 27 (11.5) | 7 (9.5) | 20 (12.4) | 0.508 |

**Supplemental Table 7.** Outcomes for Subpopulations With Pseudomonas aeruginosa as the Causative Organism Not Receiving Active Antimicrobial Therapy Prior to CAZ-AVI

|  | **Total**  ***n*=253** | **Early (<42)**  ***n*=85** | **Late (≥42)**  ***n*=168** | **p-val** |
| --- | --- | --- | --- | --- |
| **Composite Clinical Success** | 173 (68.4) | 61 (71.8) | 112 (66.7) | 0.410 |
| Absence of fever and leukocytosis 72 hrs after first CAZ/AVI dose | 220 (87.0) | 76 (89.4) | 144 (85.7) | 0.409 |
| 30-day mortality from index culture, all cause | 35 (13.8) | 10 (11.8) | 25 (14.9) | 0.498 |
| 30-day recurrence from CAZ/AVI first dose | 26 (10.3) | 8 (9.4) | 18 (10.7) | 0.747 |

**Supplemental Table 8.** Outcomes for Subpopulations With Klebsiella pneumoniae as the Causative Organism Not Receiving Active Antimicrobial Therapy Prior to CAZ-AVI

|  | **Total**  ***n*=115** | **Early (<42)**  ***n*=27** | **Late (≥42)**  ***n*=88** | **p-val** |
| --- | --- | --- | --- | --- |
| **Composite Clinical Success** | 71 (61.7) | 20 (74.1) | 51 (58.0) | 0.132 |
| Absence of fever and leukocytosis 72 hrs after first CAZ/AVI dose | 95 (82.6) | 23 (85.2) | 72 (81.8) | 0.686 |
| 30-day mortality from index culture, all cause | 24 (20.9) | 2 (7.4) | 22 (25.0) | **0.049** |
| 30-day recurrence from CAZ/AVI first dose | 10 (8.7) | 2 (7.4) | 8 (9.1) | 0.786 |
